# *Zymomonas mobilis* as an emerging biotechnological chassis for the production of industrially relevant compounds

**DOI:** 10.1186/s40643-021-00483-2

**Published:** 2021-12-16

**Authors:** Adelaide Braga, Daniela Gomes, João Rainha, Cláudia Amorim, Beatriz B. Cardoso, Eduardo J. Gudiña, Sara C. Silvério, Joana L. Rodrigues, Lígia R. Rodrigues

**Affiliations:** CEB-Centre of Biological Engineering, Universidade Do Minho, Campus de Gualtar, 4710-057 Braga, Portugal

**Keywords:** Bioproducts, Industrial chassis, Metabolic Engineering, Synthetic biology, *Zymomonas mobilis*

## Abstract

*Zymomonas mobilis* is a well-recognized ethanologenic bacterium with outstanding characteristics which make it a promising platform for the biotechnological production of relevant building blocks and fine chemicals compounds. In the last years, research has been focused on the physiological, genetic, and metabolic engineering strategies aiming at expanding *Z. mobilis* ability to metabolize lignocellulosic substrates toward biofuel production. With the expansion of the *Z. mobilis* molecular and computational modeling toolbox, the potential of this bacterium as a cell factory has been thoroughly explored. The number of genomic, transcriptomic, proteomic, and fluxomic data that is becoming available for this bacterium has increased. For this reason, in the forthcoming years, systems biology is expected to continue driving the improvement of *Z. mobilis* for current and emergent biotechnological applications. While the existing molecular toolbox allowed the creation of stable *Z. mobilis* strains with improved traits for pinpointed biotechnological applications, the development of new and more flexible tools is crucial to boost the engineering capabilities of this bacterium. Novel genetic toolkits based on the CRISPR-Cas9 system and recombineering have been recently used for the metabolic engineering of *Z. mobilis*. However, they are mostly at the proof-of-concept stage and need to be further improved.

**Graphical Abstract:**

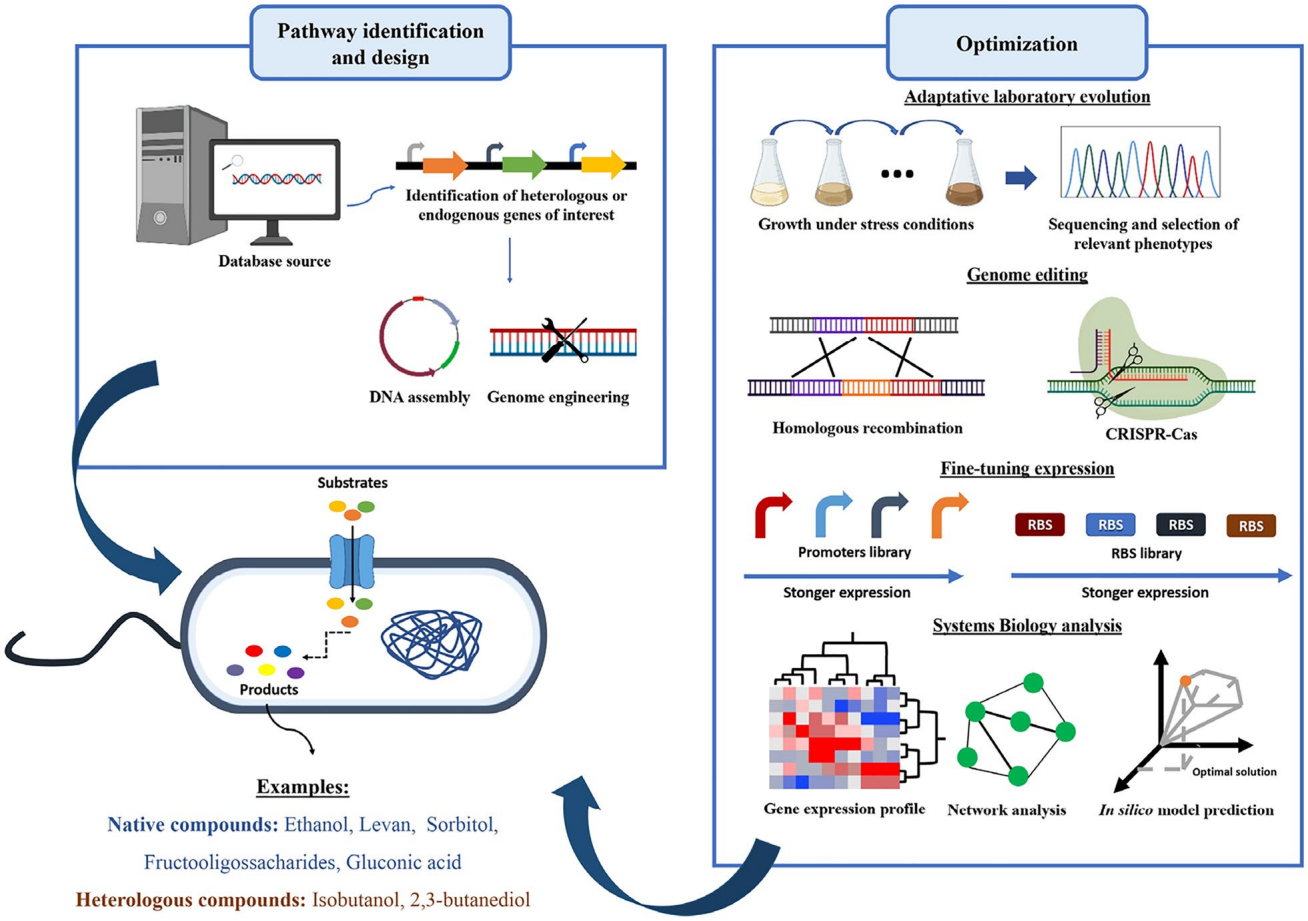

## *Zymomonas mobilis*: general overview

*Zymomonas mobilis* is a facultative anaerobic Gram-negative bacterium, that belongs to the *Sphingomonas* group of the alpha subdivision of Proteobacteria (Kosako et al. 2000). This bacterium was originally isolated from alcoholic beverages, such as the African palm wine, the Mexican “pulque,” and also as a contaminant of cider and beer in European countries (Weir 2016).

The generic name of the genus *Zymomonas* was proposed by Kluyver and Van Niel in 1936. From a taxonomical point of view, *Z. mobilis* is the unique species in the genus *Zymomonas*, and currently three subspecies have been identified: subsp. *mobilis*, subsp. *Pomaceae,* and subsp. *francensis* (Swings and De Ley 1977; Coton et al. 2006)*.* The non-model bacterium *Z. mobilis* has been gaining increased attention from the scientific community as a biotechnological workhorse in different applications (He et al. 2014; Weir 2016; Wang et al. 2018). The interest in ethanologenic bacterium stems from its tolerance to pH fluctuation (3.8 to 7.5), high sugar uptake rate, and ethanol yield. Additionally, *Z. mobilis* possesses a generally regarded as safe (GRAS) status which makes it suitable for food and pharmaceutical applications. Moreover, this bacterium is able to survive at high sugars (up to 400 g L^−1^) and ethanol (up to 160 g L^−1^) concentrations (Zhang et al. 2019a).

**Fig. 1:**
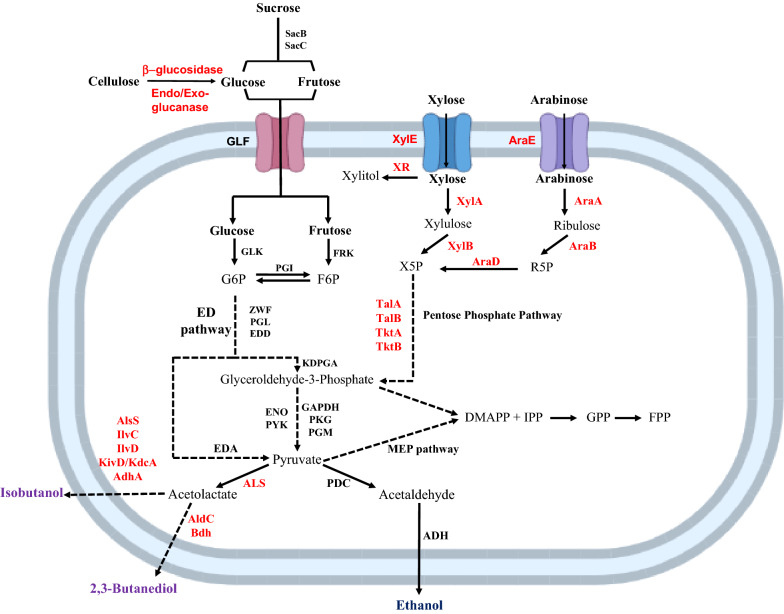
Metabolic pathways for the conversion of various sugars in *Zymomonas mobilis*. These sugars can be further used to synthesize native (blue) or heterologous (purple) value-added compounds. *ADH* alcohol dehydrogenases, *ALS* acetolactate synthase, *AlsS* acetolactate synthase S, *AldC* acetolactate decarboxylase, *AraA* arabinose isomerase; AraB: ribulokinase, *AraD* ribulose-phosphate-4-epimerase, *AraE* arabinose-proton symporter, *Bdh* butanediol dehydrogenase; *DMAPP* dimethylallyl diphosphate, *EDA* 2-keto-3-deoxy-6-phosphogluconate aldolase, *ED* Entner-Doudoroff, *EDD* 6-phosphogluconate dehydratase, *ENO* phosphopyruvate hydratase, *F6P* fructose-6-phosphate, *FPP* Farnesyl diphosphate; FRK: fructokinase, *G6P* glucose-6-phosphate, *GAPDH* glyceraldehyde-3-phosphate dehydrogenase, *GLF* glucose facilitator protein, *GLK* glucokinase; *GPP* geranyl diphosphate; *IlvC* ketol-acid reductoisomerase, *IlvD* dihydroxy-acid dehydratase, *IPP* isopentenyl diphosphate, *KdcA* alpha-ketoacid decarboxylase, *KDPGA* 2-dehydro-3-deoxy-phosphogluconate aldolase, *KivD* alpha-ketoisovalerate decarboxylase, *PDC* pyruvate dehydrogenase, *PGI* phosphoglucose isomerase, *PGL* 6-phosphogluconolactonase, *PGM* phosphoglycerate mutase, *PKG* phosphoglycerate kinase, *PYK* pyruvate kinase, *R5P* ribose-5-phosphate; *SacB* extracellular levansucrase, *SacC* extracellular sucrase; TalA: transaldolase A, *TalB* transaldolase B, *TktA* transketolase A, *TktB* transketolase B, *X5P* xylose-5-phosphate, *XR* xylose reductase, *XylA* xylose isomerase; *XylB* xylulokinase, *XylE* low-affinity xylose transporter, *ZWF* glucose-6-phosphate dehydrogenase. The endogenous metabolism is presented in black color and the heterologous enzymes expressed into *Z. mobilis* for establishing new metabolic pathways to broaden its substrate range or production of the heterologous high-value products are indicated in red. Solid lines indicate a single step; dashed lines indicate multiple steps

*Z. mobilis* is able to ferment glucose, fructose, and sucrose via Entner-Doudoroff (ED) pathway (Fig. 1), in conjugation with pyruvate decarboxylase (PDC) and two alcohol dehydrogenases (ADH), producing equimolar amounts of ethanol and carbon dioxide (Viikari and Berry 1988). PDC converts pyruvate to acetaldehyde and carbon dioxide in a non-oxidative reaction. Afterward, ADH isozymes oxidize acetaldehyde to ethanol and reduce nicotinamide adenine dinucleotide (NAD^+^) to nicotinamide adenine dinucleotide (reduced) (NADH) (Neale et al. 1987). *Z. mobilis* is the only microorganism that natively uses the ED pathway under anaerobic conditions (Viikari and Berry 1988; Kalnenieks 2006).

Figure 1

One of the most attractive features of *Z. mobilis* is its ability to produce ethanol with an outstanding yield (up to 98% of the theoretical yield) (Rogers et al. 1982). Recently, Zhang et al (2017) obtained the highest ethanol productivity reported so far, 63.7 g L^−1^ h^−1^, using polyvinyl alcohol (PVA)-immobilized cells under very high gravity (VHG) conditions with in situ ethanol removal by vacuum membrane distillation (VMD), starting from 300 g L^−1^ of glucose. Considering that this bacterium has the Pentose Phosphate (PP) pathway and the Tricarboxylic Acid (TCA) cycle incomplete, more carbon is driven into the glycolysis and ethanol production pathways, achieving an ethanol production near the theoretical maximum (Swings and De Ley 1977). This extraordinary ethanol-producing capacity is strongly related with the “uncoupled growth” phenomenon, where only 3–5% of substrate carbon is converted into biomass (Kalnenieks 2006). In fact, the biomass accumulated is three to five-fold lower when compared with the one obtained using *Escherichia coli* and *Saccharomyces cerevisiae* (Bai et al. 2008). The production of ATP by *Z. mobilis* through ED pathway is very fast and “excessive” for the cell requirements. For this reason, the presence of other ATP dissipating reactions is necessary to regenerate adenosine diphosphate (ADP) and maintain cell balance (Kalnenieks 2006). In fact, the decrease of ATP yield during alcoholic fermentation increases ethanol yield with reduced substrate conversion to biomass that can be considered a “by-product” of alcoholic fermentation. In addition, *Z. mobilis* possesses a high-specific cell surface and a higher rate of oxygen consumption, while consuming glucose faster than *S. cerevisiae* and *E. coli* (Panesar et al. 2006; Rutkis et al. 2014, 2016).

**Fig. 2:**
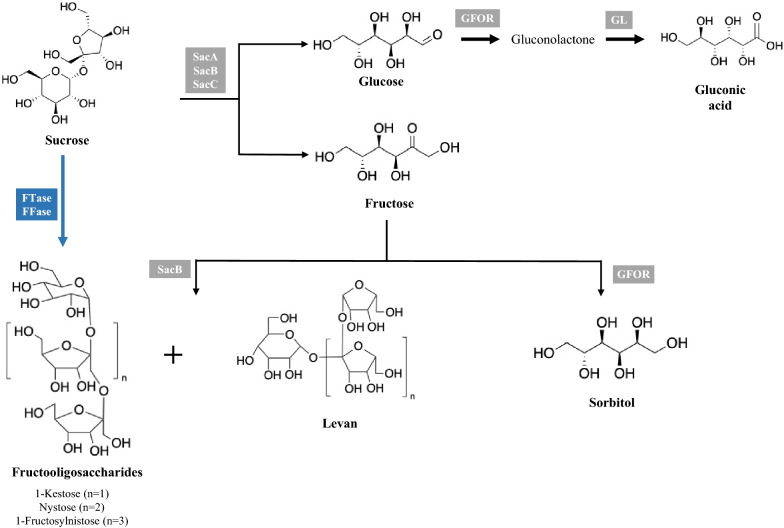
Schematic representation of biosynthetic pathways responsible for the production of fructooligosaccharides (FOS), levan, gluconic acid, and sorbitol. Black represents the *Zymomonas mobilis* native pathways. Blue represents the alternative heterologous pathways for the production of FOS. *FFase* β-fructofuranosidase, *FTase* fructosyltransferase; *GFOR* glucose–fructose oxidoreductase, *GL* gluconolactonase, *SacA* intracellular sucrose, *SacB* extracellular levansucrase; SacC: extracellular sucrase. Gray and blue boxes indicate the enzymes responsible for each conversion step

As previously mentioned, the wild-type strains of *Z. mobilis* can only consume glucose, fructose, and sucrose, as carbon source (Weir 2016). In contrast with other bacteria and yeasts, *Z. mobilis* transports sugars using a facilitated diffusion system with a glucose facilitator protein (GLF), which does not spend metabolic energy (DiMarco and Romano 1985; Snoep et al. 1994). After entering the cell, glucose is phosphorylated to glucose 6-phosphate (G6P) by glucokinase (GLK). On the other hand, fructose is phosphorylated to fructose 6-phosphate (F6P) by fructokinase (FRK). F6P can be further converted to G6P by phosphoglucose isomerase (PGI). After that, G6P is metabolized to pyruvate through the ED pathway and further converted to ethanol and carbon dioxide by PDC and ADH isozymes, as previously referred. Therefore, the ethanol yield obtained with glucose (95%) was higher than that from fructose (90%) (Viikari and Berry 1988). In addition, *Z. mobilis* is able to convert glucose to gluconic acid by glucose–fructose oxidoreductase (GFOR) and gluconolactonase (GL). Moreover, fructose is converted to sorbitol by GFOR (Barrow et al. 1984; Zachariou and Scopes 1986) (Fig. 2).

Figure 2

Sucrose is hydrolyzed to glucose and fructose either by an extracellular sucrase/invertase (SacC), intracellular sucrase (SacA) or by an extracellular levansucrase (SacB). Furthermore, SacB polymerizes fructose units to form levan, using sucrose as the fructose donor (Gunasekaran et al. 1990, 1995; Kannan et al. 1995) (Fig. 2). In fact, this bacterium only synthesizes high concentrations of levan in a sucrose medium (Johns et al. 1991). Viikari and Berry (1988) and Johns et al. (1991) reported that the synthesis of levan in *Z. mobilis* is considerably reduced in the presence of glucose and fructose (in mixtures or individually). In addition, Lyness and Doelle (1983) observed that SacB from *Z. mobilis* is completely inhibited at glucose and ethanol concentrations higher than 5.4 g L^−1^ and 73.6 g L^−1^, respectively. Besides levan, *Z. mobilis* is also able to produce FOS as a result of transfructosylation reactions during growth on sucrose (Bekers et al. 2002; Santos-Moriano et al. 2015) (Fig. 2). Doelle et al. (1990) showed that the production of FOS may be related with the deficiency of fructose uptake caused by high substrate or salt concentrations. These results suggested that high salt concentrations trigger the production of FOS instead of levan synthesis (Doelle and Doelle 1990; Doelle et al. 1990), as a response to changes in the osmotic environment (Sootsuwan et al. 2013).

In the last years, some interesting reviews have been published addressing the ecology and physiology of *Z. mobilis* (He et al. 2014; Weir 2016), as well as the recent developments on metabolic engineering strategies for bioethanol production using *Z. mobilis* (Yang et al. 2016a; Xia et al. 2019; Zhang et al. 2019a; Todhanakasem et al. 2020). Other reviews address the use of genetic tools to extend the variety of substrates that *Z. mobilis* can metabolize and the diversity of produced compounds, such as lactate, alanine, succinate, and 2,3-butanodiol (Yang et al. 2016a; Wang et al. 2018). In addition, some studies have also described the achievements and the bottlenecks of metabolic engineering of *Z. mobilis* (Wang et al. 2018).

This review presents an outline of the state-of-the-art on the use of *Z. mobilis* to synthesize an array of industrially relevant compounds along with its physiological and metabolic properties, particularly focused on the developments that have been reported in the last 5 years. In addition, we will discuss the recent available tools and methods for the genetic engineering of *Z. mobilis,* providing our point of view mainly related to the handling and genetic manipulation of *Z. mobilis*. Moreover, we will also review the work developed to expand the substrate utilization by this bacterium. Finally, we will present an overview of the strategies that are being used to produce native and heterologous compounds by *Z. mobilis*.

## Genetic engineering tools available for *Z. mobilis*

As previously mentioned, attributable to its physiological and metabolic characteristics, *Z. mobilis* appears as an attractive host for the development of microbial cell factories to industrially produce relevant or high-value biological compounds. Related to the development of efficient cell factories is the need to modify wild strains in order to maximize or silence a specific phenotype. The synthetic biology field offers the possibility to rapidly modify the genetic elements of a certain organism allowing a predictable phenotypic response by the engineered organism.

**Fig. 3:**
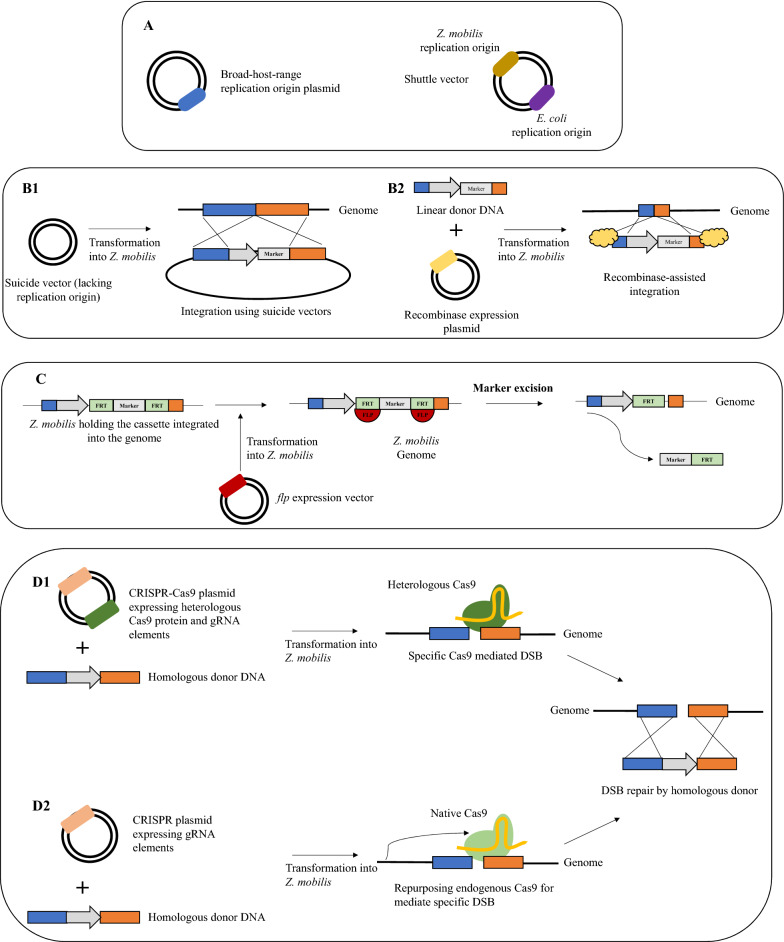
Genetic engineering tools to modify *Zymomonas mobilis*: **A** Plasmid-based approaches: Plasmids containing broad-host range replication origins can be maintained in *Z. mobilis* or shuttle vectors having *Z. mobilis* replication origin and an *E. coli* replication origin can be used instead; **B** Genome integration can be achieved in *Z. mobilis* via homologous recombination (HR) using a suicide vector, that is a plasmid lacking a suitable replication origin, or by using a plasmid expressing recombinases to catalyze HR and linear DNA fragments as donor template; **C** Genome editing mediated by Clustered Regularly Short Palindromic Repeats-associated Cas (CRISPR-Cas) systems: Heterologous CRISPR-Cas9 systems can be efficiently expressed in *Z. mobilis* to generate a double strand break (DSB) in genome followed by repair by HR by donor template. Here, a plasmid carrying a heterologous Cas9 protein and the guide RNA (gRNA) components is co-transformed with the homologous donor DNA fragment. Otherwise, the endogenous CRISPR-Cas system can be programmed to produce a specific DSB to be repaired by HR. Thereunto, a plasmid only carrying the gRNA elements is co-transformed with the homologous donor DNA fragment

The attempts to genetically modify *Z. mobilis* started using plasmid vectors (Carey et al. 1983; Browne et al. 1984; Conway et al. 1987). *E. coli*–*Z. mobilis* shuttle vectors, which contain replicons from *E. coli* and from *Z. mobilis* native plasmids, are currently the most popular plasmid-based expression systems (So et al. 2014) (Fig. 3A).

Researchers noticed that the introduction of foreign DNA is the main complication to genetically modify this bacterium. Chemical transformation or electroporation of plasmids or other DNA parts into *Z. mobilis* is very inefficient. In fact, *Z. mobilis* contains an highly active type I and type IV DNA endogenous restriction-modification (R-M) system that is able to efficiently degrade foreign DNA, thus explaining the low transformation efficiencies usually observed in this microorganism (Dong et al. 2011; Kerr et al. 2011; Felczak et al. 2021). The type I system acts primarily on unmethylated DNA, while the type IV system acts primarily on methylated DNA. Regardless, Zou et al. (2012b) described a procedure to enhance *Z. mobilis* electrotransformation efficiency. The authors observed significantly higher transformation efficiencies when using type I R-M inhibitor and unmethylated plasmid DNA. Indeed, our own experience supports this evidence since we found that the source of bacterial plasmid DNA is critical for an efficient *Z. mobilis* electrotransformation. We observed a significantly higher transformation efficiency when the plasmids were previously replicated in *E. coli* JM110 (*dam*-/*dcm*-), a strain used to isolate unmethylated DNA. Another issue in the construction of shuttle vectors is the selection markers available. We tested the antibiotic sensitivities of ZM4 strain as an initial step for genetic studies using *Z. mobilis*. The resistance of the strain was tested against kanamycin, ampicillin, chloramphenicol, spectinomycin, and tetracycline. *Z. mobilis* ZM4 is highly resistant to kanamycin and ampicillin (up to 200 and 700 µg mL^−1^, respectively). On the other hand, this bacterium was not able to grow at 100 µg mL^−1^ of streptomycin and chloramphenicol, and it was sensitive to tetracycline at concentrations above 25 μg mL^−1^. In fact, these antibiotics are the most commonly used with *Z. mobilis*. However, even using these antibiotics, false positives during transformation processes often occur (*unpublished data*).

The first attempts to modify the *Z. mobilis* genome started to be performed using transposable elements on a suicide vector, that lacks a suitable replication origin (Fig. 3B1) (Pappas et al. 1997). However, for efficient recombineering, several homology base pairs are required (higher than 300 bp), and the transformation efficiencies are low. Despite that, this technique has been widely used (Senthilkumar et al. 2004; Kalnenieks et al. 2006; Wang et al. 2012). More recently, new methods used to engineer bacterial chromosomal genes have been implemented in *Z. mobilis*. Wu et al. (2017) demonstrated the use of Enterobacteriophage RecT system to perform direct genes knockout. Using this method, only 60 bp homology regions were required for integration. Moreover, Khandelwal et al. (2018) used the bacteriophage lambda Red genes system to assist direct genomic modifications on the bacterium genome. A homologous region of 40 bp was enough to perform recombination. These methods use recombinases to catalyze the homologous recombination process allowing the use of linear DNA molecules with shorter homology regions as donor templates. This avoids the need to construct a vector since donor DNA oligos can be generated by a single PCR reaction. In these cases, the linear homologous fragment and a plasmid carrying the recombinase machinery are co-transformed into *Z. mobilis* (Fig. 3B2)*.* After recombination, recombinases can be used to catalyze further genomic modifications by transforming another linear donor DNA. Alternatively, the plasmid can be cured. Both recombinase-assisted methods, as well as the integration methods using suicide vectors are associated with the introduction of antibiotic markers to select positive mutants. As previously mentioned, the natural resistance of *Z. mobilis* to several antibiotics (Bochner et al. 2010) hampers the selection of positive mutants and limits the number of modifications that can be performed. To overcome that, the *S. cerevisiae* flippase recognition target (FRT)- flippase(flp) recombination system was also applied in *Z. mobilis* for in vivo marker excision (Fig. 3C) (Zou et al. 2012a). The combination of this system with bacteriophage lambda recombination system may be used for multiple genetic modifications by recycling the antibiotic marker. In addition, more recently, Lal et al. (2019) reported for the first time a markerless method for genome engineering by homologous recombination using suicide vectors. The suicide vectors were introduced via conjugation carrying a 500 bp homology flanking the genetic region of interest.

The implementation of a synthetic biology tool capable of circumventing the use of large homology arms and antibiotics to perform genomic modifications represents an advantage for genetic engineers. The application of Clustered Regularly Interspaced Short Palindromic Repeats CRISPR-associated protein (CRISPR-Cas) system for genetic engineering purposes is a landmark in the synthetic biology field (Rainha et al. 2021). It allows to perform high-efficient, rapid and markerless genetic manipulations since it eliminates the requirement of using antibiotic markers to select positive mutants which represents a great advantage when dealing with *Z. mobilis*. Hereupon, CRISPR-mediated genome engineering in *Z. mobilis* can be performed using a heterologous CRISPR-Cas system, such as the one from *Streptococcus pyogenes*. For that purpose, a vector expressing Cas protein and the gRNA elements are co-transformed with a linear donor DNA carrying the desired modification (Fig. 3D1). Researchers postulated that *Z. mobilis* may lack a non-homologous end joining function to heal the double breakage. For this reason, the break is repaired by providing a linear homologous donor DNA carrying the modification. The first work reporting the application of CRISPR-Cas system in *Z. mobilis* was published by Cao et al. (2017). The researchers used the CRISPR-Cas9 system of *S. pyogenes* to knockout the replicase genes of *Z. mobilis* and eliminate native plasmids. Most of the established CRISPR-Cas systems for genetic modifications relies on the use of type II nucleases, such as the one from *S. pyogenes*, that were found to be toxic in certain bacteria (Zhang and Voigt 2018). In this sense, Shen et al. (2019) used Cas12a from *Francisella novicida*, a type V endonuclease, with reported less toxicity to prokaryotes.

Like other bacteria, *Z. mobili*s possesses a native CRISPR system as an immune defense mechanism against foreign DNA (Zheng et al. 2019). Dong et al*.* (Dong et al. 2016) characterized and classified the *Z. mobilis* endogenous type I CRISPR system. By providing the specific RNA elements it is possible to program *Z. mobilis* native type I CRISPR-Cas system to generate a specific break in the genome. The use of the native CRISPR-Cas may be advantageous because the possible toxicity of a heterologous Cas protein can be avoided. The repurposing of native *Z. mobilis* CRISPR-Cas system has already been employed to perform single and multiplex gene knockout, gene insertion, gene replacement, and single point mutations (Fig. 3D2) (Zheng et al. 2019). Due to its advantages, the use of endogenous CRISPR-Cas systems to genetically modify *Z. mobilis* has been registered in some patents (Lixin et al. 2019a, b).

In addition to the use of CRISPR to perform genome modifications, novel CRISPR applications, such as CRISPR interference (CRISPRi) technology (Banta et al. 2020), have also been applied in *Z. mobilis*. CRISPRi is an extremely useful methodology to study gene functions and to fine-tune gene expression since it uses programmable guide RNAs and Cas proteins to perform knockdowns in a controlled and measurable way. In a first attempt to use this technique, Z. *mobilis* Cas 2/3 was modified to be used as native CRISPRi system. However, its knockdown efficiency was very low (Zheng et al. 2019). For this reason, Banta et al. (2020) have constructed a new CRISPRi methodology for *Z. mobilis* (Mobile-CRISPRi system) based on the heterologous *S. pyogenes* dCas9 system, improving the knockdown efficacy.

The selection of proper genetic elements is also a crucial step for metabolic engineering purposes. The non-coding genetic elements include promoters, ribosome binding sites (RBS), untranslated regions, and terminators. For example, Yang et al. (2019) successfully developed a reporter system to identify candidate promoters with different strengths using data from omics datasets. The promoters Pgap, Ppdc, and Peno were classified as strong, being Pgap the strongest. Additionally, they identified medium and weak strength novel promoters. In total, 38 promoters and 4 RBSs were characterized. However, a limited small number of native promoters have been employed until now.

Despite synthetic biology studies have been initiated early in *Z. mobilis*, the development and implementation of novel techniques to perform genetic modifications remains little explored when compared with other biological chassis, such as *E. coli*. The non-amenability to insert foreign DNA may have hampered the application of such techniques. However, as previously mentioned, due to its metabolic characteristics, *Z. mobilis* represents an attractive microorganism for industrial engineering purposes, increasing the research interest in the development of new methodologies to genetically improve this chassis.

## Use of alternative substrates and feedstock

Wild-type *Z. mobilis* needs a fermentable sugar to grow. Naturally, this microorganism is unable to consume pentoses, such as arabinose and xylose, and complex sugars, such as starch and cellulose (Swings and De Ley 1977). The growing concerns about the use of carbon sources that compete with food supply production, associated with global warming and environmental degradation, promote the exploitation of renewable and inexpensive feedstock. Sustainable feedstock should include, for instance, waste lignocellulose derived from agriculture and forestry residues and by-products from fruit and vegetable processing processes. Currently, the application of different metabolic engineering approaches allowed the development of engineered *Z. mobilis* strains able to cost-efficiently convert renewable biological resources and waste streams into valuable products (Braide et al. 2018; Sarkar et al. 2020).

Lignocellulosic wastes are the most abundant renewable source of sugars, and their hydrolysis produce a mixture of sugars that includes hexoses (glucose, mannose, and galactose) and pentoses (xylose and arabinose) (Fig. 1). In the last years, several approaches have been developed to produce ethanol using lignocellulosic materials as substrate*.* Zhang et al. (1995) reported for the first time the construction of a recombinant *Z. mobilis* strain able to metabolize xylose to ethanol by introducing a combination of the non-oxidative portion of PP pathway and xylose assimilation pathway genes in the wild-type strain. The obtained strain was able of growing on xylose with a specific growth rate of 0.057 h^−1^ and an ethanol yield of 86%. Since then, efforts have been made to construct recombinant strains able to consume pentose sugars by heterologous expression of xylose and arabinose metabolism. Adaptive Laboratory Evolution (ALE) strategies—process of achieving mutations under specific selection pressure—combined with direct metabolic engineering, have also been adopted to improve the co-utilization of sugars by *Z. mobilis* (Agrawal et al. 2011; Mohagheghi et al. 2015; Sarkar et al. 2020). Last year, Sarkar et al. (2020) reported an ALE approach to develop a *Z. mobilis* strain with efficient co-utilization of glucose and xylose. For that purpose, a recombinant xylose fermenting strain was cultivated under selective pressure of increasing xylose concentration (from 30 to 100 g L^−1^). After 50 transfers, the selected genetically modified strain showed a 1.6-fold increase in xylose utilization rate. The results of this study demonstrated that the observed phenotypic response may be related with enhanced activity of xylose isomerase (XylA), that catalyzes the conversion of xylose to xylulose; upregulated transketolase activity (encoded by *tkt*); and downregulated xylose reductase (XR) activity (encoded by *xyrA*). ‬‬‬‬‬‬‬‬‬‬‬‬‬

In addition to genetic engineering approaches to produce ethanol from lignocellulosic residues, other strategies have also been used. The most common technologies are simultaneous saccharification and fermentation (SSF)—a one-step enzymatic hydrolysis and fermentation—and separate hydrolysis and fermentation (SHF)—a sequential enzymatic hydrolysis of cellulose and hemicellulose and their further fermentation. However, the pretreatments required to hydrolyze lignocellulosic feedstocks into monosaccharides comprise an undesirable step that leads to the formation of lignocellulose-derived by-products that inhibit microbial biocatalysts in SHF. Another interesting and alternative approach that has been recently reported is the co-fermentation of hexoses and pentoses from lignocellulosic hydrolysates. Co-culture strategies have been highlighted as an interesting and alternative strategy for the utilization of C5 and C6 sugars by *Z. mobilis* (Fu and Peiris 2008; Fu et al. 2009; Nguyen et al. 2019). Dewi et al. (2019) studied ethanol production from sugar palm (*Arenga pinnata*) using a co-culture of *Z. mobilis* and *Pichia stipitis*. The first strain was able to ferment glucose to ethanol with high yields; the second can naturally produce ethanol from xylose. This co-culture system produced 0.57 g g^−1^ of ethanol. Recently*,* Wirawan et al. (2020) used *Z. mobilis* immobilized in PVA and *P. stipitis* (suspended cells). The authors obtained a higher ethanol yield in separate hydrolysis and co-fermentation (SHCF) process (0.414 g g^−1^, 81.7% of theoretical yield) when compared with simultaneous saccharification and co-fermentation (SSCF) (0.36 g g^−1^, 70.65% of theoretical yield). Nevertheless, the cellulose enzymatic hydrolysis was required. In order to improve the utilization of lignocellulosic materials, several cellulolytic-encoding genes have been cloned and expressed in *Z. mobilis* (Yoon et al. 1988; Brestic-Goachet et al. 1989; Vasan et al. 2011; Jung et al. 2012; Luo and Bao 2015). A consolidated bioprocess (CBP) is a promising competitive approach for bioethanol production from lignocellulosic hydrolysates. In this process, a microorganism is able to produce saccharolytic enzymes to degrade polysaccharides (cellulose or hemicelluloses) from lignocellulosic materials into fermentable sugars that are further used to produce ethanol. However, these studies only demonstrated lignocellulosic materials conversion in resting cells. In order to overcome this issue, Kurumbang et al. (2020) proposed the heterologous expression and secretion of a glycosyl hydrolase (GH) β-glucosidase from *Caulobacter crescentus* in *Z. mobilis*, that enables an efficient conversion of oligosaccharides. The engineered strain was further subjected to an adaptation in cellobiose medium, and growth on cellobiose was achieved. However, the authors observed an increased lag phase in cellobiose medium. Nevertheless, the simultaneous expression of cellulases and xylanases plays an important role in degrading lignocellulose into fermentable sugars.

Besides lignocellulosic feedstock, other wastes and residues have been used as substrate by *Z. mobilis,* including sugarcane, sweet sorghum, carob, sugar beet, waste paper sludge, sweet potato, bamboo residues, sweet sorghum stalk, corncob residues, sugarcane molasses, rice bran, as well as algal biomass from *Spirogyra hyaline* (Behera et al. 2012; He et al. 2013; Saharkhiz et al. 2013; Ma et al. 2016; Sulfahri et al. 2016)*.* The ethanol production from agricultural wastes (cassava, yam and potato peels) using *Z. mobilis* was evaluated by Braide et al. (2018), that reported ethanol yields that ranged from 5.17 to 8.36% (v/v). The use of *Z. mobilis* biofilms was also highlighted as an interesting approach to overcome the problems associated with toxic inhibitors (such as acetic acid, furfural, organic acids) that are present in some wastes and residues (Todhanakasem et al. 2014). Ma’As et al. (2020) reported the production of ethanol by *Z. mobilis* ATCC 31,821 using Brewer’s rice, which is composed of 80% of starch, as substrate. In this study, 9.67 g L^−1^ of ethanol was produced after 22 h of fermentation. The same substrate was also fermented by *S. cerevisiae* ATCC 200,062 and a lower ethanol production (4.31 g L^−1^) was obtained. This result shows the potential of *Z. mobilis* to be used as biocatalyst for ethanol production from alternative substrates.

The development of *Z. mobilis* as a viable platform host to produce industrially relevant compounds is strongly dependent on using renewable and inexpensive feedstock. In fact, the production of cellulosic ethanol using this microorganism is already implemented at a commercial scale (Yang et al. 2016a).

## Production of added-value compounds by *Z. mobilis*

*Z. mobilis* holds the biochemical pathways responsible to produce several interesting compounds. In addition, *Z. mobilis* has proven to be a suitable host for the heterologous production of several added-value products. In this section, the production of native compounds (e.g., ethanol, levan, FOS, sorbitol and gluconic acid) and heterologous compounds (e.g., 2,3-butanediol and isobutanol) in *Z. mobilis* will be reviewed. In this topic, we will present a summary of the recent advances (last 5 years) in the production of these different industrial relevant compounds by *Z. mobilis*, focusing not only on the genetic enhancement, when appropriate, but also on the production process, comparing the used carbon source and cultivation strategies (Table 1).

**Table 1:** Relevant compounds produced in *Zymomonas mobilis* (studies performed in the last 5 years), and its industrialization status

Product	Strain^a^	Substrate/precursor	Condition (pH, temperature)	Fermentation strategy^b^	Titer (g L^−1^)	Productivity (g L^−1^ h^−1^)	Reference	Industrialization status
Ethanol	TMY-FHPX	Xylose	pH 6 32 °C	Batch-VHG	136	2.3	(Wang et al. 2016)	DuPont implemented an industrial scale process for cellulosic bioethanol production from corn stover, currently licensed to China and Macedonia
	DSM 473	Glucose	pH 5.5 30 °C	Batch	50	0.40	(Palamae et al. 2020)	
				2 cycle repeated batch	46	0.68		
				3 cycle repeated batch	60	0.64		
	ZMA7-2	Glucose	30 °C	Microplate	50	0.83	(Shui et al. 2015)	
	ZMA7-3				50	0.83		
	ZMA7-2	Food waste	pH 4 30 °C	Bench scale	100	2.3	(Ma et al. 2016)	
				Flask scale	98	2.1		
	ZMA-142	Glucose + sodium acetate	pH 5 30 °C	Bench scale	61	1.7	(Liu et al. 2017)	
	ZMA-167				47	0.99		
	ATCC 29501	Glucose	pH 5.5 35 °C	Batch-VHG	75	4.7	(Ajit et al. 2017)	
	ATCC 29501	Glucose	pH 7 room temperature	PVA-immobilized cells VHG + VMD	127	6	(Zhang et al. 2017)	
	8b	Lignocellulosic hydrolysates	pH 5.8 30 °C	VHG	35	1.8	(Li et al. 2019)	
Levan	CCT 4494	Sucrose	pH 7 30 °C	Continuous fermentation with immobilized cells	113	6.3	(Lorenzetti et al. 2015)	Real Biotech Co started an industrial process
	CCT 4494	Sucrose	pH 4 30 °C	Repeated batch with immobilized cells	21	0.88	(Santos and Cruz 2016)	
Levan (cont.)	CCT 4494	Sucrose	pH 7 40 °C	Repeated batch with immobilized cells	32	1.3	(Santos and Cruz 2017)	
	PTCC 1718	Sucrose	28 °C	Flask scale	57	1.2	(Taran et al. 2019)	
Sorbitol and Gluconic acid (GA**)**	ATCC 29501	Fructose + Glucose	pH 6.4 39 °C	Bench scale (200 mL)	66 (sorbitol) 70 (GA)	2.7 (sorbitol) 2.9 (GA)	(Folle et al. 2018)	Low Technology readiness levels (TRL) Weakness: simultaneous production of two products Solution: development of an efficient and cost-effective separation and purification process Strengths: produced at high titers
	ZM4 *gnl*Δ	Fructose + Glucose	pH 6 30 °C	BiotransformationFlask scale	65 (sorbitol) 67 (GA)	2.7 (sorbitol) 2.8 (GA)	(Alvin et al. 2017)	
2,3-butanodiol	9C	Glucose + Xylose	pH 5.8 30 °C	Flask scale	15	0.31	(Yang et al. 2016b)	Low TRL Weakness: early stage of development Strengths: GRAS microorganism; reduced aeration requirement; limited by-product formation
		Glucose			13	0.54		
	Zmo-BDOI	Glucose + Xylose	pH 5.8 30 °C	Fed-batch	120	0.13	(Zhang et al. 2019b)	
Isobutanol	Zmo-IBA1	Glucose	pH 6 30 °C	Batch in STR with gas stripping	6	0.08	(Liu et al. 2020b)	Low TRL Weakness: early stage of development Strengths: GRAS microorganism; abundant pyruvate biosynthesis; limited by-product formation
	ZMQ3-A4	Glucose	30 °C	Flask scale	4	0.33	(Qiu et al. 2020)	

^a^TMY-FHPX: CP4 derivative strain that heterologous express xylose isomerase, xylulokinase, transaldolase, and transketolase and *yfdZ*/*metB* operon from *E. coli,* thioesterase from *Acinetobacter baylyi*, fatty acyl-CoA reductases from jojoba, and wax ester synthase/acyl-CoA–diacyl glycerol acyltransferase from *A. baylyi*; ZMA7-2 and ZMA7-3: mutated version of ZM4 by adaptive laboratory evolution (ALE) with high tolerance to acetic acid; ZMA-142 and ZMA-167: mutated versions of ATCC31823 by ALE and chemical mutagenesis with high tolerance to acetate; 9C: 8b derivative strain with chloramphenicol and tetracycline resistance genes cured; ZM4 *gnl*Δ: ZM4 derivative strain with gluconolactonase inactivated and chloramphenicol resistance genes cured; Zmo-BDOI: modified version of 9C strain. This strain was constructed by integration of the genes responsible for 2,3-butanediol biosynthetic pathway in a strain with the pyruvate decarboxylase (PDC) gene deleted (Zmo-pdcI strain); *Zmo-IBA1* modified version of 9C strain. The strain was constructed by integration of two plasmids holding the isobutanol biosynthetic genes in Zmo-pdcI strain, *ZMQ3-A4* ZM4 derivative strain through integration of *kdcA* gene from *Lactococcus lactis* in *Z. mobilis* genome and expression of the isobutanol biosynthetic genes. ^**b**^*VHG* very high gravity; *PVA* polyvinyl alcohol, *VMD* vacuum membrane distillation, *STR* stirred-tank reactor

### Native products

#### Ethanol

In the last years, the use of *Z. mobilis* as a microbial cell factory to produce ethanol has been extensively reviewed (Rogers et al. 2007; He et al. 2014; Yang et al. 2016a; Xia et al. 2019; Zhang et al. 2019a; Todhanakasem et al. 2020). As previously described, metabolic engineering methodologies have been explored to construct *Z. mobilis* strains able to use xylose or arabinose to produce ethanol. For example, Grisales Díaz and Willis (2019) have developed a kinetic model to study the co-fermentation of xylose and glucose toward the production of ethanol. Unlike the models previously proposed by Leksawasdi et al. (2001) and Hodge and Karim (2002), the Grisales Díaz and Willis (2019) model considers the production of xylitol which is a compound that inhibits the xylose utilization. By co-fermentation of both compounds, the model predicted that ethanol production could reach 90 g L^−1^. When xylose is the only substrate, the ethanol production was estimated to reach 70 g L^−1^. With this study it was possible to perform a more accurate in silico estimation of ethanol production given that it is considered the xylitol production and its inhibitory effect on xylose consumption and, consequently, on ethanol production.

The production of high concentrations of ethanol is also significantly affected by different factors, such as inhibitors (ethanol, acetic acid, furfural, among others), low pH, and osmotic and oxidative stress (Zhang et al. 2019a). Wang et al. (2019) used the genome shuffling technology in order to enhance *Z. mobilis* tolerance to furfural and acetic acid. Genome shuffling consists in the recombination of the genome of selected strains (parental strains) combined with the electrofusion of protoplasts. Using this methodology, more tolerant strains with high productivity can be obtained. In this study, two parental strains AQ8-1 and F34 were subjected to two rounds of genome shuffling. After these rounds, 10 mutants were selected due to their tolerance to 5 g L^−1^ of acetic acid and 3 g L^−1^ of furfural. Within these mutants, two of them were selected as promising ones since they showed higher ethanol productivities comparing to the parental strains when incubated with both inhibitors. The study of genes involved in the stress response in *Z. mobilis* could be also a useful strategy to improve the tolerance to these toxic inhibitors. Nouri et al. (2020) have studied the effect of the overexpression of *hfq* and *sigE* that encode a transcription regulator and a transcription factor involved in furfural and acetic acid stress responses, respectively. The overexpression of both genes resulted in higher ethanol production and higher tolerance to inhibitors comparing to the wild-type strain. However, the overexpression of *sigE* led to highest ethanol production levels in the presence of inhibitors comparing with the strains overexpressing *hfq* or both genes. This study demonstrated that the genes involved in the response of *Z. mobilis* to stress could be relevant targets to improve the tolerance to inhibitors. Another interesting approach that could be explored is the conversion of these inhibitors in less aggressive compounds in a process called biodetoxification. Yi et al. (2019) constructed an oxidative pathway in order to perform the conversion of the toxic phenolic aldehydes while ethanol is produced. The oxidative pathway was constructed by expressing benzaldehyde dehydrogenase from *Pseudomonas putida* and overexpressing NADH-dependent alcohol dehydrogenase from *Z. mobilis*. The expression of both genes resulted in the complete conversion of some toxic compounds, as well as in the improvement of ethanol fermentability. Alternatively, Liu et al. (2020a) also overexpressed genes encoding cofactors related to oxidoreductase in order to manipulate the intracellular redox in *Z. mobilis* and, consequently, increase the tolerance to inhibitors. In this study, it was found that oxidoreductases could be directly involved in the biodetoxification of furfural. Moreover, the modified strains with higher ATP levels and lower concentration of reactive oxygen species were found to be more tolerant to sodium acetate and sodium formate. This result suggests that the intracellular redox is an important mechanism to control and improve the tolerance of these strains to inhibitors. All these studies demonstrated that these stress-tolerant strains have potential to be used in the production of ethanol at an industrial level. However, these strains should be uninterruptedly modified toward the development of tolerance to multiple stress factors leading to the construction of even more robust, tolerant, and efficient strains (Zhang et al. 2019a). In fact, the production of ethanol using *Z. mobilis* can be competitive compared with other microorganisms. The highest ethanol production (127.4 g L^−1^) was obtained with *Z. mobilis* ATCC 29,191 cells using VHG + VMD fermentation, with a yield that reached 85% of the theoretical maximum (0.51 g g^−1^) (Zhang et al. 2017). Moreover, this titer was higher than the highest value reported in the well-recognized ethanol producer *S. cerevisiae* (114.71 g L^−1^) (Wu et al. 2020)*.* Using *Z. mobilis* to produce ethanol is advantageous compared to *S. cerevisiae* and *E. coli* since less than 50% of biomass is accumulated during fermentation (Zhao et al. 2014). This occurs because sugars are fueled for ethanol production instead of being used for biomass accumulation.

#### Levan

Beyond its ability to produce ethanol, *Z. mobilis* is also able to produce levan. Levan is a natural polymer composed of fructose residues linked by β(2 → 6) glycosidic bonds and presents multiple β(2 → 1)-linked branching points (Öner et al. 2016). *Z. mobilis* can produce SacB enzyme that is responsible for levan and FOS production using sucrose as substrate (Bekers et al. 2002; Santos-Moriano et al. 2015) (Fig. 2). In the last years, the production of levan using *Z. mobilis* as microbial cell factory was widely studied. Taran et al. (2019) conducted a statistical optimization to determine the growth conditions that improve the production of levan by *Z. mobilis* PTCC 1718. The effect of sucrose, yeast extract, and potassium phosphate concentrations were determined. When the fermentation was performed in a medium containing 300 g L^−1^ of sucrose, 1 g L^−1^ of yeast extract, and 0.5 g L^−1^ of potassium phosphate, 57 g L^−1^ of levan was produced. Another strategy explored to produce levan was the use of immobilized *Z. mobilis* cells. Santos and Cruz evaluated the production of levan by *Z. mobilis* CCT4494 cells immobilized on alginate and chitosan beads (Santos and Cruz 2016). Using sequential batch fermentation, 22.11 g L^−1^ of levan was obtained. The same authors also evaluated the levan production by immobilized *Z. mobilis* CCT4494 cells on sugarcane bagasse and loofa sponge. The highest production of levan was obtained when sugarcane bagasse was used as immobilization support in the sequential batch fermentations, resulting in the production of 32.13 g L^−1^ of levan (Santos and Cruz 2017). Moreover, the production of levan was also evaluated in continuous fermentation by immobilized *Z. mobilis* CCT4494 cells in hybrid system of alginate/PVA. Using an initial sucrose concentration of 300 g L^−1^ and initial pH of 7.0, 112.53 g L^−1^ of levan was obtained after 18 h of fermentation, with a yield (0.375 g g^−1^) near to the theoretical maximum (0.395 g g^−1^) (Lorenzetti et al. 2015). Nowadays, levan is already produced at an industrial scale by Real Biotech Co., Ltd., Chungnam, Korea, using the levansucrase from *Z. mobilis* (Öner et al. 2016).

#### Fructooligosaccharides (FOS)

Beyond levan, FOS are also interesting prebiotic compounds that are natively produced in Z*. mobilis*. These compounds are carbohydrates composed of fructose residues with a terminal glucose molecule residue linked by β(2 → 1) glycosidic bonds (Flores-Maltos et al. 2016). As already mentioned, FOS are produced from sucrose by *Z. mobilis* SacB enzyme (Bekers et al. 2002; Santos-Moriano et al. 2015; Erdal et al. 2017; Taştan et al. 2019) (Fig. 2). In 2015, our group reported a co-culture strategy that consists in the use of *Aureobasidium pullulans* to produce FOS, followed by the use of *S. cerevisiae* or *Z. mobilis* to reduce the concentration of monosaccharides present in the culture medium (Nobre et al. 2015). This two-step fermentation process was used to obtain higher yields of purified FOS. Using *Z. mobilis* in the second step of the fermentations, the total percentage of FOS increased from 56% (one-step fermentation with *A. pullulans*) to 81%. More recently, we have been exploring the potential of *Z. mobilis* to produce relevant amounts of FOS in a simple one-step fermentation bioprocess. Under static conditions, 30 g L^−1^ of FOS (1-kestose, nystose, and 6-kestose) was produced using 300 g L^−1^ of sucrose as substrate. However, the obtained yield is far behind the theoretical maximum (0.5–0.65 g g^−1^). Moreover, 5.8 g L^−1^ of levan, 18 g L^−1^ of sorbitol, and 50 g L^−1^ of ethanol were also produced (Braga et al. 2019). This study demonstrated the potential of a faster and sustainable process for simultaneous production of FOS, sorbitol, and levan using *Z. mobilis* ZM4 as a whole-cell biocatalyst. Other microorganisms, such as *A. pullulans,* are able to produce FOS through transfructosylation of sucrose by fructosyltransferase enzyme (FTase)/β-fructofuranosidase (FFase) (Khanvilkar and Arya 2015). For this reason, we are also exploring the possibility of overexpressing a FTase/FFase from a recognized fungal source using metabolic engineering methodologies as an alternative and promising approach to increase the production of FOS by *Z. mobilis* in a one-stage bioprocess. With this approach it is possible to avoid the drawbacks of two-stage processes, which starts with the microbial enzyme production followed by a second stage that includes the incubation of the extracted enzyme with sucrose to produce FOS. Using the one-step approach, we expect to produce the enzyme and FOS subsequently in the same bioreactor.

Although *Z. mobilis* exhibits a high potential to produce FOS, this microorganism has not been widely explored for this purpose. Several *Aspergillus* species are recognized as FOS producers and a lot of work has been developed using these species (de la Rosa et al. 2019). For example, we reported that *Aspergillus ibericus* produced 118 g L^−1^ of total FOS from 200 g L^−1^ of sucrose in a one-step process (Nobre et al. 2018). Despite the residual use of *Z. mobilis* as FOS producer, we believe that this microorganism is an interesting alternative cell factory to produce prebiotics in a single-step fermentation, since it can convert the non-prebiotic sugars (glucose and fructose) into added-value products, such as levan, sorbitol and ethanol, avoiding competitive inhibition of the fructosyl transfer reaction. Nevertheless, there is still a long way to go before production levels can be competitive as compared to other producers.

#### Sorbitol and gluconic acid

*Z. mobilis* can also naturally produce sorbitol and gluconic acid when grown on sucrose or on a mixture of fructose and glucose (Barrow et al. 1984; Leigh et al. 1984) (Fig. 2)*.* The metabolic pathway responsible for the production of both compounds was first proposed by Leigh et al*.* (Leigh et al. 1984) that reported two enzymes attached by an unidentified cofactor capable of oxidizing glucose to gluconolactone and reducing fructose to sorbitol. Two years later, this enzyme was described as GFOR by Zachariou and Scopes (1986), which uses NADPH and NADP to reduce fructose to sorbitol and to oxidize glucose to gluconolactone, respectively. Afterward, GL hydrolyzes gluconolactone into gluconic acid (Zachariou and Scopes 1986). Research efforts have been made in order to develop sorbitol and gluconic acid-producing processes with high yields and titers (Rogers et al. 2007; He et al. 2014). One of the most common approach used to increase the sorbitol and gluconic acid production is the use of permeabilized cells. This methodology allowed the inactivation of the fermentative metabolism of *Z. mobilis*, since metallic ions and cofactors diffuse out of the cells inactivating the pathway from gluconic acid to ethanol (Chun and Rogers 1988; Rehr et al. 1991). However, the use of non-permeabilized cells to achieve the production of sorbitol and gluconic acid was also proposed to avoid operational limitations and decrease the costs of the production of these compounds in a large scale (Silveira et al. 1999; Folle et al. 2018). A new approach was presented by Folle et al. (2018). The authors proposed the use of glutaraldehyde as a reticulation agent instead of cetyltrimethylammonium bromide, allowing a simple procedure to prepare calcium alginate beads, without a treatment prior to immobilization. Afterward, they compared the sorbitol production in mechanically and pneumatically stirred reactors, in batch and fed-batch mode. A high sorbitol concentration (around 137 g L^−1^) was obtained in mechanically and pneumatically stirred reactors using the fed-batch operation mode. Another issue in the production of sorbitol at a biotechnological level is the relative high cost of the substrates, particularly pure fructose. In order to overcome this, the use of alternative and cheap feedstock has been studied. For example, An et al. (2013) proposed an interesting approach for sorbitol and gluconic acid production using cassava starch and inulin that are considered low-cost feedstocks. The authors used a commercial glucoamylase enzyme for saccharification of cassava starch and inulin into glucose and fructose, replacing the expensive inulinase enzyme. The obtained fructose and glucose were used to produce sorbitol and gluconic acid with sodium alginate/PVA-immobilized whole cells of the *Z. mobilis* strain, reaching to sorbitol and gluconic acid titers of 180 g L^−1^ and 193 g L^−1^, respectively. Furthermore, understanding the physiological function of all the enzymes involved in the biosynthetic pathway could be useful to construct a microorganism with improved characteristics. The physiological function of GL that converts gluconolactone into gluconic acid was studied by Alvin et al. (2017). Sorting to genetic engineering approaches, the authors have constructed a *Z. mobilis* ZM4 with a knockout in the GL-encoding gene. The studies performed with the mutant strain demonstrated that the gene encoding GL enzyme is not necessary for the maintenance of the strain. Moreover, all the gluconolactone that was produced in the fermentation process was fully converted to gluconic acid (67 g L^−1^ from 100 g L^−1^ of glucose and 100 g L^−1^ of fructose) even without GL activity, due to a spontaneous conversion mechanism. The mutant strain was also subjected to several stress conditions to understand the physiological function of GL. This experiment found that the mutant strain was more susceptible to stress conditions than the parental strain being possible to conclude that GL can be involved in the anti-stress responses of *Z. mobilis* contributing to its industrial robustness (Alvin et al. 2017). Beyond *Z. mobilis*, only a few microorganisms were identified as natural (Tani and Vongsuvanlert 1987; Duvnjak et al. 1991) or genetically modified sorbitol producers (Nissen et al. 2005; Jan et al. 2017). For example, *Candida boidinii* produced 19.1 g L^−1^ of sorbitol from 20 g L^−1^ of fructose (Tani and Vongsuvanlert 1987). Additionally, 9.78 g L^−1^ of sorbitol was produced by *Lactobacillus plantarum* (Jan et al. 2017). Gluconic acid is also naturally produced by other microorganisms (Roukas 2000; Anastassiadis et al. 2003; Ahmed et al. 2015). For example, 71.85 g L^−1^ of gluconic acid was produced by *A. niger* (Ahmed et al. 2015)*.* Still, the titers reported with *Z. mobilis* are among the highest levels reported so far. These studies clearly show that *Z. mobilis* could be an excellent platform to produce sorbitol and gluconic acid at an industrial scale, with a conversion rate of almost 100%. However, to replace the chemical industrial process by a fermentation-based biotechnological one, it will be necessary to overcome some weaknesses, such as the simultaneously production of the two products and the development of an efficient and cost-effective separation and purification process. Moreover, it will be important to prevent the production of the by-product ethanol in order to increase the production yields of these compounds.

### Heterologous products

In addition to the compounds of interest that are naturally produced in *Z. mobilis*, there are several compounds that this microorganism does not produce naturally that are very interesting at an industrial level (Fig. 1).

#### 2,3-Butanediol

The 2,3-butanediol is considered a platform bulk chemical with interesting industrial applications in the production of several chemical feedstock, liquid fuel additives, perfumes, pharmaceuticals, and food additives (Hazeena et al. 2020). The metabolic pathway responsible for the production of 2,3-butanediol was already engineered in *Z. mobilis*. To construct this biosynthetic pathway, three genes from *Bacillus licheniformis* and *Bacillus subtilis* encoding acetolactate synthase (ALS), acetolactate decarboxylase (AldC), and butanediol dehydrogenase (Bdh) were introduced into *Z. mobilis* 8b. In this study, it was found that all the three genes are essential for 2,3-butanediol production in *Z. mobilis*, reaching more than 10 g L^−1^ of 2,3-butanediol produced using glucose and xylose as starter substrates (Yang et al. 2016b). More recently, the heterologous production of this compound was successfully improved by constructing a *Z. mobilis* strain expressing the 2,3-butanediol biosynthetic pathway and holding a knockout in the PDC gene. Using the fed-batch operation mode, the obtained strain was able to produce 120 g L^−1^ of 2,3-butanediol from a total 650 g L^−1^ fed of glucose and xylose (Zhang et al. 2019b). The 2,3-butanediol biosynthetic pathway was also explored to evaluate the capacity of *Z. mobilis* strains to grow aerobically without the PDC gene. To investigate this, the authors have expressed the PDC gene under the control of an IPTG-inducible promoter (LacI promoter) in a strain with this gene deleted, constructing the *Zmo-pdcI* strain. Additionally, the 2,3-butanediol biosynthetic pathway genes were expressed under the control of a promoter inducible by anhydrotetracycline (aTc) (TetR promoter) in the *Zmo-pdcI* strain, constructing the *Zmo-*BDOI strain. The ability of this strain to grow aerobically or anaerobically, when only the 2,3-butanediol pathway was expressed, was assessed. The authors determined that the strain without PDC gene expression only has the capacity to grow aerobically and the expression of 2,3-butanediol biosynthetic pathway was sufficient for the recycling of NADH (Liu et al. 2020b).

Despite all the efforts that have been developed in the last years, the yield of 2,3-butanediol (0.18 g g^−1^) obtained was significantly lower than the theoretical maximum reported (0.52 g g^−1^). However, *Z. mobilis* can be an interesting industrial host to produce 2,3-butanediol due to its GRAS status, since most of the native producers of this compound belong to the risk group 2 of organisms (*Klebsiella* sp., *Enterobacter* sp., *Pseudomonas* sp., *Serratia* sp.). Furthermore, these microorganisms produce a combination of stereoisomers, which require additional purification steps to achieve pure stereoisomers. *E. coli* and *S. cerevisiae* have already been engineered to produce 2,3-butanediol, with titers ranging from 73.8 to 154.3 g L^−1^, respectively (Xu et al. 2014; Kim et al. 2016). Nevertheless, *Z. mobili*s has several advantages, namely, the high sugar uptake and consequently low biomass production, and minimized aeration need, lowering the production costs (Yang et al. 2016b).

#### Isobutanol

Isobutanol is considered a promising next-generation biofuel that could replace gasoline due to its lower hygroscopicity and higher energy density (Chen and Liao 2016). To produce this compound in *Z. mobilis*, the *Z. mobilis Zmo-pdcI* strain was transformed with two plasmids carrying the five genes responsible for isobutanol biosynthetic pathway (*alsS* from *B. subtilis* that encodes an acetolactate synthase; *ilvC* from *E. coli* encoding ketol-acid reductoisomerase; dihydroxy-acid dehydratase, encoded by *ilvD,* from *Z. mobilis*; and *kivD* and *adhA* from *Lactococcus lactis,* that encodes an alpha-ketoisovalerate decarboxylase and alcohol dehydrogenase, respectively) under the control of an aTc-inducible promoter. After induction with aTc and IPTG, the strain produced 32 mM (2.37 g L^−1^) of isobutanol. Since this amount of isobutanol could be toxic to the cells, the authors have removed isobutanol from the cultures using the N_2_ gas-stripping system and the evaporated isobutanol was collected with a cooling condenser. Using this strategy, the isobutanol production reached 80 mM (6 g L^−1^) from 170 mM of glucose (Liu et al. 2020b). The isobutanol biosynthetic pathway was also constructed in *Z. mobilis* ZM4 by Qiu et al. (2020). Since *ilvC* and *ilvD* are involved in the valine synthesis in *Z. mobilis*, these native genes were overexpressed in conjugation with the expression of *alsS* from *B. subtilis* and alpha-ketoacid decarboxylase, encoded by *kdcA*, from *L. lactis.* This strain produced 4.01 g L^−1^ of isobutanol from 45 g L^−1^ of glucose. Moreover, it was found that the production of ethanol decreased in this strain, being possible to conclude that the carbon flux responsible for ethanol production was directed to isobutanol production. However, the yields obtained in both studies are far from the theoretical maximum (0.41 g g^−1^).

Beyond *Z. mobilis*, the production of isobutanol has been reported in several microorganisms (*Corynebacterium glutamicum* (Smith et al. 2010; Blombach et al. 2011)*, S. cerevisiae* (Chen et al. 2011)). Nevertheless, most of these processes require aeration which increases the production costs. Moreover, it is difficult to prevent the by-products formation and maintain the redox balance. These issues can be overcome using *Z. mobilis* since it contains an anaerobic ED pathway, a truncated TCA cycle and a metabolism that allows high pyruvate formation maintaining the redox balancing and limiting the by-products formation (Buijs et al. 2013; Liu et al. 2016; Morita et al. 2017; Ghosh et al. 2019).

## Conclusion

*Z. mobilis* has emerged as a very promising microbial platform to produce biofuels and other relevant biomolecules from renewable feedstock, due to its unique characteristics. In fact, the production of cellulosic ethanol using *Z. mobilis* is already implemented at a commercial scale. In 2015, the former DuPont opened a commercial scale biorefinery in Nevada, Iowa (USA), that produces cellulosic bioethanol from corn stover using a recombinant *Z. mobilis* strain developed by the National Renewable Energy Laboratory (USA). DuPont started to produce 30 million gallons of fuel ethanol per year; however, it was sold in 2017. This decision comes from the challenges associated with oil prices and political uncertainty. On the other hand, the use of waste materials in large-scale industrial processes implies assuring a reliable constant supply and usually a pretreatment step that may add a significant cost to the process. Regardless of the progress made in the last decades on the production of building blocks and fine chemical compounds using *Z. mobilis*, some bottlenecks ought to be overcome to make the industrial production of these compounds a reality*.* The industrialization of these compounds is dependent on the yields and the productivities obtained. In general, to implement an industrial process, the yield should be at least 85% of the theoretical maximum and the productivity must be around 2 g L^−1^ h^−1^ (Peralta-Yahya et al. 2012; Woodley 2017). To enable this, the challenges that still need to be addressed are the limited substrate range of this microorganism and the disruption of competing pathways. Additionally, its highly active restriction-modification system has limited the development of new and efficient genetic tools to engineer this organism for many years. In fact, redirecting the *Z. mobilis* metabolism from ethanol fermentation pathway toward the production of other added-value compounds remains a bottleneck. However, the recent advances in genetic engineering strategies and the application of novel synthetic biology approaches, such as CRISPR-Cas, rational strain engineering, and ALE, will allow the establishment of *Z. mobilis* as an alternative microorganism to produce non-native bioproducts.

Recently, *Z. mobilis* was successfully engineered to allow a flexible metabolic control, namely through a control switch for specific ethanol-producing enzymes. This kind of approach could be used in a near future to obtain different *Z. mobilis* strains capable of synthesizing other bioproducts than ethanol. Furthermore, with current industry trends suggesting that cellulosic ethanol may not be the fuel of the future, research efforts will focus on the potential of *Z. mobilis* to produce fatty acids, sorbitol, gluconic acid, 2,3-butanodiol, and levan. Real Biotech Co., Ltd., Chungnam, Korea, for instance, has already launched a process for levan production with *Z. mobilis.*

Although the production of these industrial relevant compounds in *Z. mobilis* is being explored, there is still a long way to go regarding its economic and environmentally friendly industrial production. Therefore, it is also crucial to optimize the fermentation conditions, including culture media and operating parameters, as well as the product extraction and purification methodologies. Nevertheless, all these studies support the idea that *Z. mobilis* is a microorganism with great potential to be used as biocatalyst for ethanol production at an industrial scale. Moreover, it is necessary to develop new pilot-scale processes with this bacterium to demonstrate its superiority over strains, such as *S. cerevisiae,* to produce specific compounds at an industrial level.

## Data Availability

The datasets (graphs and tables) supporting the conclusions of this article are available.

## Abbreviations

ADH: Alcohol dehydrogenases
ADP: Adenosine diphosphate
ALE: Adaptive Laboratory Evolution
ALS: Acetolactate synthase
AlsS: Acetolactate synthase S
AldC: Acetolactate decarboxylase
AraA: Arabinose isomerase
AraB: Ribulokinase
AraD: Ribulose-phosphate-4-epimerase
AraE: Arabinose-proton symporter
aTc: Anhydrotetracycline
ATP: Adenosine triphosphate
Bdh: Butanediol dehydrogenase
CBP: Consolidated bioprocess
CRISPR-Cas9: Clustered Regularly Short Palindromic Repeats-associated protein 9
DMAPP: Dimethylallyl diphosphate
DSB: Double-strand break
DNA: Deoxyribonucleic acid
ED: Entner-Doudoroff
EDA: 2-Keto-3-deoxy-6-phosphogluconate aldolase
EDD: 6-Phosphogluconate dehydratase
ENO: Phosphopyruvate hydratase
F6P: Fructose-6-phosphate
FLP: Flippase
FOS: Fructooligosaccharides
FFase: β-Fructofuranosidase
FPP: Farnesyl diphosphate
FTase: Fructosyltransferase enzyme
FRT: Flippase recognition target
FRK: Fructokinase
G6P: Glucose-6-phosphate
GAPDH: Glyceraldehyde-3-phosphate dehydrogenase
GFOR: Glucose–fructose oxidoreductase
GH: Glycosyl hydrolase
GL: Gluconolactonase
GLF: Glucose facilitator protein
GLK: Glucokinase
GPP: Geranyl diphosphate
gRNA: Guide ribonucleic acid
IlvC: Ketol-acid reductoisomerase
IlvD: Dihydroxy-acid dehydratase
IPTG: Isopropyl ß-D-1-thiogalactopyranoside
IPP: Isopentenyl diphosphate
KdcA: Alpha-ketoacid decarboxylase
KDPGA: 2-Dehydro-3-deoxy-phosphogluconate aldolase
KivD: Alpha-ketoisovalerate decarboxylase
PCR: Polymerase chain reaction
PDC: Pyruvate dehydrogenase
PGI: Phosphoglucose isomerase
PGL: 6-Phosphogluconolactonase
PGM: Phosphoglycerate mutase
PP: Pentose Phosphate
PKG: Phosphoglycerate kinase
PYK: Pyruvate kinase
PVA: Polyvinyl alcohol
NAD +: Nicotinamide adenine dinucleotide
NADH: Nicotinamide adenine dinucleotide
R5P: Ribose-5-phosphate
RBS: Ribosome binding sites
R-M: Restriction-modification
SacA: Intracellular sucrase
SacB: Extracellular levansucrase
SacC: Extracellular sucrase
SHCF: Separate hydrolysis and co-fermentation
SHF: Separate hydrolysis and fermentation
SSF: Saccharification and fermentation
STR: Stirred-tank reactor
TalA: Transaldolase A
TalB: Transaldolase B
TCA: Tricarboxylic Acid
TktA: Transketolase A
TktB: Transketolase B
VMD: Vacuum membrane distillation
VHG: Very high gravity
X5P: Xylose-5-phosphate
XR: Xylose reductase
XylA: Xylose isomerase
XylB: Xylulokinase
XylE: Low-affinity xylose transporter
ZWF: Glucose-6-phosphate dehydrogenase

## References

[CR1] Agrawal M, Mao Z, Chen RR (2011). Adaptation yields a highly efficient xylose-fermenting *Zymomonas mobilis* strain. Biotechnol Bioeng.

[CR2] Ahmed AS, Farag SS, Hassan IA, Botros HW (2015). Production of gluconic acid by using some irradiated microorganisms. J Radiat Res Appl Sci.

[CR3] Alvin A, Kim J, Jeong GT (2017). Industrial robustness linked to the gluconolactonase from *Zymomonas mobilis*. Appl Microbiol Biotechnol.

[CR4] An K, Hu F, Bao J (2013). Simultaneous saccharification of inulin and starch using commercial glucoamylase and the subsequent bioconversion to high titer sorbitol and gluconic acid. Appl Biochem Biotechnol.

[CR5] Anastassiadis S, Aivasidis A, Wandrey C (2003). Continuous gluconic acid production by isolated yeast-like mould strains of *Aureobasidium pullulans*. Appl Microbiol Biotechnol.

[CR6] Bai FW, Anderson WA, Moo-Young M (2008). Ethanol fermentation technologies from sugar and starch feedstocks. Biotechnol Adv.

[CR7] Banta A, Enright A, Siletti C, Peters JM (2020). A high-efficacy CRISPR interference system for gene function discovery in *Zymomonas mobilis*. Appl Environ Microbiol.

[CR8] Barrow K, Collins J, Leigh D (1984). Sorbitol production by *Zymomonas mobilis*. Appl Microbiol Biotechnol.

[CR9] Behera S, Mohanty RC, Ray RC (2012). Ethanol fermentation of sugarcane molasses by *Zymomonas mobilis* MTCC 92 immobilized in *Luffa cylindrica* L. sponge discs and ca-alginate matrices. Brazilian J Microbiol.

[CR10] Bekers M, Laukevics J, Upite D (2002). Fructooligosaccharide and levan producing activity of *Zymomonas mobilis* extracellular levansucrase. Process Biochem.

[CR11] Blombach B, Riester T, Wieschalka S (2011). *Corynebacterium glutamicum* tailored for efficient isobutanol production. Appl Environ Microbiol.

[CR12] Bochner B, Gomez V, Ziman M (2010). Phenotype microarray profiling of *Zymomonas mobilis* ZM4. Appl Biochem Biotechnol.

[CR13] Braga A, Amorim C, Rodrigues JL et al (2019) *Zymomonas mobilis* as a whole-cell biocatalyst for the production of prebiotics. In: Abstracts of MicroBiotec 19 - Congress of Microbiology and Biotechnology 2019. University of Coimbra, Coimbra, Portugal, 5–7 Dec 2019, p 482

[CR14] Braide W, Oji IO, Adeleye SA, Korie MC (2018). Comparative study of bioethanol production from sugarcane molasses by using *Zymomonas mobilis* and *Saccharomyces cerevisiae*. Int J Appl Microbiol Biotechnol Res.

[CR15] Brestic-Goachet N, Gunasekaran P, Cami B, Baratti JC (1989). Transfer and Expression of an *Erwinia chrysanthemi* Cellulase Gene in *Zymomonas mobilis*. Microbiology.

[CR16] Browne GM, Skotnicki ML, Goodman AE, Rogers PL (1984). Transformation of *Zymomonas mobilis* by a hybrid plasmid. Plasmid.

[CR17] Buijs NA, Siewers V, Nielsen J (2013). Advanced biofuel production by the yeast *Saccharomyces cerevisiae*. Curr Opin Chem Biol.

[CR18] Cao QH, Shao HH, Qiu H (2017). Using the CRISPR/Cas9 system to eliminate native plasmids of *Zymomonas mobilis* ZM4. Biosci Biotechnol Biochem.

[CR19] Carey VC, Walia SK, Ingram LO (1983). Expression of a lactose transposon (Tn951) in *Zymomonas mobilis*. Appl Environ Microbiol.

[CR20] Chen CT, Liao JC (2016). Frontiers in microbial 1-butanol and isobutanol production. FEMS Microbiol Lett.

[CR21] Chen X, Nielsen KF, Borodina I (2011). Increased isobutanol production in *Saccharomyces cerevisiae* by overexpression of genes in valine metabolism. Biotechnol Biofuels.

[CR22] Chun UH, Rogers PL (1988). The simultaneous production of sorbitol from fructose and gluconic acid from glucose using an oxidoreductase of *Zymomonas mobilis*. Appl Microbiol Biotechnol.

[CR23] Conway T, Byun M, Ingram L (1987). Expression Vector for *Zymomonas mobilis*. Appl Environ Microbiol.

[CR24] Coton M, Laplace JM, Auffray Y, Coton E (2006). Polyphasic study of *Zymomonas mobilis* strains revealing the existence of a novel subspecies *Z. mobilis* subsp*. francensis* subsp. nov., isolated from French cider. Int J Syst Evol Microbiol.

[CR25] de la Rosa O, Flores-Gallegos AC, Muñíz-Marquez D (2019). Fructooligosaccharides production from agro-wastes as alternative low-cost source. Trends Food Sci Technol.

[CR26] Dewi AS, Stevanus RA, Sandra MA (2019). The effect of mixed culture of *zymomonas mobilis* and *pichia stipitis* in ethanol production of sugar palm (*Arenga pinnata*). Mater Sci Forum.

[CR27] Díaz VHG, Willis MJ (2019). Ethanol production using *Zymomonas mobilis*: Development of a kinetic model describing glucose and xylose co-fermentation. Biomass Bioenerg.

[CR28] DiMarco AA, Romano AH (1985). D-Glucose transport system of *Zymomonas mobilis*. Appl Environ Microbiol.

[CR29] Doelle MB, Doelle HW (1990). Sugar-cane molasses fermentation by *Zymomonas mobilis*. Appl Microbiol Biotechnol.

[CR30] Doelle MB, Greenfield PF, Doelle HW (1990). The relationship between sucrose hydrolysis, sorbitol formation and mineral ion concentration during bioethanol formation using *Zymomonas mobilis* 2716. Appl Microbiol Biotechnol.

[CR31] Dong HW, Bao J, Ryu DDY, Zhong JJ (2011). Design and construction of improved new vectors for *Zymomonas mobilis* recombinants. Biotechnol Bioeng.

[CR32] Dong G, He M, Feng H (2016). Functional characterization of CRISPR-Cas system in the ethanologenic bacterium *Zymomonas mobilis* ZM4. Adv Microbiol.

[CR33] Duvnjak Z, Turcotte G, Duan ZD (1991). Production of sorbitol and ethanol from Jerusalem artichokes by *Saccharomyces cerevisiae* ATCC 36859. Appl Microbiol Biotechnol.

[CR34] Erdal Ö, Kaplan-Türköz B, Taştan Ö, Göksungur Y (2017). Levansucrase production by *Zymomonas mobilis*: Optimization of process parameters and fructooligosaccharide production. J Food Biochem.

[CR35] Felczak MM, Bowers RM, Woyke T, Teravest MA (2021). Biotechnology for Biofuels *Zymomonas* diversity and potential for biofuel production. Biotechnol Biofuels.

[CR36] Flores-Maltos DA, Mussatto SI, Contreras-Esquivel JC (2016). Biotechnological production and application of fructooligosaccharides. Crit Rev Biotechnol.

[CR37] Folle AB, Baschera VM, Vivan LT (2018). Assessment of different systems for the production of aldonic acids and sorbitol by calcium alginate-immobilized *Zymomonas mobilis* cells. Bioprocess Biosyst Eng.

[CR38] Fu N, Peiris P (2008). Co-fermentation of a mixture of glucose and xylose to ethanol by *Zymomonas mobilis and Pachysolen tannophilus*. World J Microbiol Biotechnol.

[CR39] Fu N, Peiris P, Markham J, Bavor J (2009). A novel co-culture process with *Zymomonas mobilis* and *Pichia stipitis* for efficient ethanol production on glucose/xylose mixtures. Enzyme Microb Technol.

[CR40] Ghosh IN, Martien J, Hebert AS (2019). OptSSeq explores enzyme expression and function landscapes to maximize isobutanol production rate. Metab Eng.

[CR41] Gunasekaran P, Karunakaran T, Cami B (1990). Cloning and sequencing of the *sacA* gene: Characterization of a sucrase from *Zymomonas mobilis*. J Bacteriol.

[CR42] Gunasekaran P, Mukundan G, Kannan R (1995). The *sacB* and *sacC* genes encoding levansucrase and sucrase form a gene cluster in *Zymomonas mobilis*. Biotechnol Lett.

[CR43] Hazeena SH, Sindhu R, Pandey A, Binod P (2020). Lignocellulosic bio-refinery approach for microbial 2,3-Butanediol production. Bioresour Technol.

[CR44] He M, Li Q, Liu X (2013). Bio-ethanol production from bamboo residues with lignocellulose fractionation technology (LFT) and separate hydrolysis fermentation (SHF) by *Zymomonas Mobilis*. Am J Biomass Bioenergy.

[CR45] He MX, Wu B, Qin H (2014). *Zymomonas mobilis*: a novel platform for future biorefineries. Biotechnol Biofuels.

[CR46] Hodge DB, Karim MN (2002). Modeling and advanced control of recombinant *Zymomonas mobilis* fed-batch fermentation. Biotechnol Prog.

[CR47] Jan KN, Tripathi AD, Singh S (2017). Enhanced sorbitol production under submerged fermentation using *Lactobacillus plantarum*. Appl Food Biotechnol.

[CR48] Johns MR, Greenfield PF, Doelle HW, Fiechter A (1991). Byproducts from *Zymomonas mobilis*. Advances in Biochemical Engineering/Biotechnology.

[CR49] Jung SK, Parisutham V, Jeong SH, Lee SK (2012). Heterologous expression of plant cell wall degrading enzymes for effective production of cellulosic biofuels. J Biomed Biotechnol.

[CR50] Kalnenieks U (2006). Physiology of *Zymomonas mobilis*: Some Unanswered Questions. Adv Microb Physiol.

[CR51] Kalnenieks U, Galinina N, Toma MM (2006). Respiratory behaviour of a *Zymomonas mobilis adhB::kanr* mutant supports the hypothesis of two alcohol dehydrogenase isoenzymes catalysing opposite reactions. FEBS Lett.

[CR52] Kannan R, Mukundan G, Aït-Abdelkader N (1995). Molecular cloning and characterization of the extracellular sucrase gene (*sacC*) of *Zymomonas mobilis*. Arch Microbiol.

[CR53] Kerr AL, Jeon YJ, Svenson CJ (2011). DNA restriction-modification systems in the ethanologen, *Zymomonas mobilis* ZM4. Appl Microbiol Biotechnol.

[CR54] Khandelwal R, Agrawal S, Singhi D (2018). Deletion of pyruvate decarboxylase gene in *Zymomonas mobilis* by recombineering through bacteriophage lambda red genes. J Microbiol Methods.

[CR55] Khanvilkar SS, Arya SS (2015). Fructooligosaccharides: Applications and health benefits: a review. Agro Food Ind Hi Tech.

[CR56] Kim JW, Kim J, Seo SO (2016). Enhanced production of 2,3-Butanediol by engineered *Saccharomyces cerevisiae* through fine-tuning of Pyruvate decarboxylase and NADH oxidase activities. Biotechnol Biofuels.

[CR57] Kosako Y, Yabuuchi E, Naka T, et al (2000) Proposal of Sphingomonadaceae fam. nov., consisting of Sphingomonas Yabuuchi et al. 1990, Erythrobacter shiba and shimidu 1982, Erythromicrobium Yurkov et al. 1994, Porphyrobacter Fuerst et al. 1993, Zymomonas Kluyver and van Niel 1936, and Sandaracinobac. Microbiol Immunol 44:563–575. doi: 10.1111/j.1348-0421.2000.tb02535.x 10.1111/j.1348-0421.2000.tb02535.x 10981829

[CR58] Kurumbang NP, Vera JM, Hebert AS (2020). Heterologous expression of a glycosyl hydrolase and cellular reprogramming enable *Zymomonas mobilis* growth on cellobiose. PLoS ONE.

[CR59] Lal PB, Wells FM, Lyu Y (2019). A Markerless Method for Genome Engineering in *Zymomonas mobilis* ZM4. Front Microbiol.

[CR60] Leigh D, Scopes RK, Rogers PL (1984). A proposed pathway for sorbitol production by Zymomonas mobilis. Appl Microbiol Biotechnol.

[CR61] Leksawasdi N, Joachimsthal EL, Rogers PL (2001). Mathematical modelling of ethanol production from glucose/xylose mixtures by recombinant *Zymomonas mobilis*. Biotechnol Lett.

[CR142] Li Y, Zhai R, Jiang X, Chen X, Yuan X, Liu Z, Jin M (2019). Boosting ethanol productivity of *Zymomonas mobilis *8b in enzymatic hydrolysate of dilute acid and ammonia pretreated corn stover through medium optimization high cell density fermentation and cell recycling. Front Microbiol..

[CR62] Liu Z, Liu P, Xiao D, Zhang X (2016). Improving isobutanol production in metabolically engineered *Escherichia coli* by co-producing ethanol and modulation of pentose phosphate pathway. J Ind Microbiol Biotechnol.

[CR141] Liu YF, Hsieh CW, Chang YS, Wung BS (2017) Effect of acetic acid on ethanol production by *Zymomonas mobilis* mutant strains through continuous adaptation. BMC Biotechnol 17(1). 10.1186/s12896-017-0385-y 10.1186/s12896-017-0385-y PMC5540488 28764759

[CR63] Liu CG, Cao LY, Wen Y (2020). Intracellular redox manipulation of *Zymomonas mobilis* for improving tolerance against lignocellulose hydrolysate-derived stress. Chem Eng Sci.

[CR64] Liu Y, Ghosh IN, Martien J (2020). Regulated redirection of central carbon flux enhances anaerobic production of bioproducts in *Zymomonas mobilis*. Metab Eng.

[CR65] Lixin M, Wenfang P, Shihui Y, et al (2019a) The efficient delet method of genome large fragment and its application based on the endogenous CRISPR-Cas system of zymomonas mobilis. CN110408642A.

[CR66] Lixin M, Wenfang P, Shihui Y, et al (2019b) Edit methods and its application simultaneously of polygenic locus based on the endogenous CRISPR-Cas system of *Zymomonas mobilis.* CN110331158A.

[CR67] Lorenzetti MFS, Moro MR, García-Cruz CH (2015). Alginate/PVA beads for levan production by *Zymomonas mobilis*. J Food Process Eng.

[CR68] Luo Z, Bao J (2015). Secretive expression of heterologous β-glucosidase in *Zymomonas mobilis*. Bioresour Bioprocess.

[CR69] Lyness EW, Doelle H (1983). Levansucrase from *Zymomonas mobilis*. Biotechnol Lett.

[CR70] Ma’As MF, Ghazali HM, Chieng S (2020). Bioethanol production from Brewer’s rice by Saccharomyces cerevisiae and *Zymomonas mobilis*: evaluation of process kinetics and performance. Energy Sources Part A Recover Util Environ Eff.

[CR71] Ma K, Ruan Z, Shui Z (2016). Open fermentative production of fuel ethanol from food waste by an acid-tolerant mutant strain of *Zymomonas mobilis*. Bioresour Technol.

[CR72] Mohagheghi A, Linger JG, Yang S (2015). Improving a recombinant *Zymomonas mobilis* strain 8b through continuous adaptation on dilute acid pretreated corn stover hydrolysate. Biotechnol Biofuels.

[CR73] Morita K, Nomura Y, Ishii J (2017). Heterologous expression of bacterial phosphoenol pyruvate carboxylase and Entner-Doudoroff pathway in *Saccharomyces cerevisiae* for improvement of isobutanol production. J Biosci Bioeng.

[CR74] Neale AD, Scopes RK, Wettenhall REH, Hoogenraad NJ (1987). Pyruvate decarboxylase of *Zymomonas mobilis*: Isolation, properties, and genetic expression in *Escherichia coli*. J Bacteriol.

[CR75] Nguyen DTT, Praveen P, Loh KC (2019). Co-culture of *Zymomonas mobilis* and *Scheffersomyces stipitis* immobilized in polymeric membranes for fermentation of glucose and xylose to ethanol. Biochem Eng J.

[CR76] Nissen L, Pérez-Martínez G, Yebra MJ (2005). Sorbitol synthesis by an engineered *Lactobacillus casei* strain expressing a sorbitol-6-phosphate dehydrogenase gene within the lactose operon. FEMS Microbiol Lett.

[CR77] Nobre C, Castro CC, Hantson A-L (2015). Production of High-Content Fructo-Oligosaccharides. World Acad Sci Eng Technol Int J Nutr Food Eng.

[CR78] Nobre C, Alves Filho EG, Fernandes FAN (2018). Production of fructo-oligosaccharides by *Aspergillus ibericus* and their chemical characterization. LWT - Food Sci Technol.

[CR79] Nouri H, Moghimi H, Marashi SA, Elahi E (2020). Impact of *hfq* and *sigE* on the tolerance of *Zymomonas mobilis* ZM4 to furfural and acetic acid stresses. PLoS ONE.

[CR80] Öner ET, Hernández L, Combie J (2016). Review of Levan polysaccharide: From a century of past experiences to future prospects. Biotechnol Adv.

[CR139] Palamae S, Choorit W, Chatsungnoen T, Chisti Y (2020). Simultaneous nitrogen fixation and ethanol production by Zymomonas mobilis. J Biotechnol.

[CR81] Panesar PS, Marwaha SS, Kennedy JF (2006). *Zymomonas mobilis*: An alternative ethanol producer. J Chem Technol Biotechnol.

[CR82] Pappas KM, Galani I, Typas MA (1997). Transposon mutagenesis and strain construction in *Zymomonas mobilis*. J Appl Microbiol.

[CR83] Peralta-Yahya PP, Zhang F, Del Cardayre SB, Keasling JD (2012). Microbial engineering for the production of advanced biofuels. Nature.

[CR84] Qiu M, Shen W, Yan X (2020). Metabolic engineering of *Zymomonas mobilis* for anaerobic isobutanol production. Biotechnol Biofuels.

[CR85] Rainha J, Rodrigues JL, Rodrigues LR (2021). CRISPR-Cas9: A powerful tool to efficiently engineer *Saccharomyces cerevisiae*. Life.

[CR86] Rehr B, Wilhelm C, Sahm H (1991). Production of sorbitol and gluconic acid by permeabilized cells of *Zymomonas mobilis*. Appl Microbiol Biotechnol.

[CR87] Rogers PL, Lee KJ, Skotnicki ML, Tribe DE (1982). Ethanol production by *Zymomonas mobilis*. Microbial Reactions.

[CR88] Rogers PL, Jeon YJ, Lee KJ, Lawford HG (2007). *Zymomonas mobilis* for fuel ethanol and higher value products. Adv Biochem Eng Biotechnol.

[CR89] Roukas T (2000). Citric and gluconic acid production from fig by *Aspergillus niger* using solid-state fermentation. J Ind Microbiol Biotechnol.

[CR90] Rutkis R, Galinina N, Strazdina I, Kalnenieks U (2014). The inefficient aerobic energetics of *Zymomonas mobilis*: Identifying the bottleneck. J Basic Microbiol.

[CR91] Rutkis R, Strazdina I, Balodite E (2016). The low energy-coupling respiration in *Zymomonas mobilis* accelerates flux in the entner-doudoroff pathway. PLoS ONE.

[CR92] Saharkhiz S, Mazaheri D, Shojaosadati SA (2013). Evaluation of bioethanol production from carob pods by *Zymomonas mobilis* and *Saccharomyces cerevisiae* in solid submerged fermentation. Prep Biochem Biotechnol.

[CR93] Santos-Moriano P, Fernandez-Arrojo L, Poveda A (2015). Levan versus fructooligosaccharide synthesis using the levansucrase from *Zymomonas mobilis*: effect of reaction conditions. J Mol Catal B Enzym.

[CR94] Santos VAQ, Cruz CHG (2016). Ethanol and levan production by sequential bath using *Zymomonas mobilis* immobilized on alginate and chitosan beads. Acta Sci - Technol.

[CR95] Santos VAQ, Cruz CHG (2017). *Zymomonas mobilis* immobilized on loofa sponge and sugarcane bagasse for levan and ethanol production using repeated batch fermentation. Brazilian J Chem Eng.

[CR96] Sarkar P, Mukherjee M, Goswami G, Das D (2020). Adaptive laboratory evolution induced novel mutations in *Zymomonas mobilis* ATCC ZW658: a potential platform for co-utilization of glucose and xylose. J Ind Microbiol Biotechnol.

[CR97] Senthilkumar V, Rameshkumar N, Busby SJW, Gunasekaran P (2004). Disruption of the *Zymomonas mobilis* extracellular sucrase gene (*sacC*) improves levan production. J Appl Microbiol.

[CR98] Shen W, Zhang J, Geng B (2019). Establishment and application of a CRISPR-Cas12a assisted genome-editing system in *Zymomonas mobilis*. Microb Cell Fact.

[CR140] Shui ZX, Qin H, Wu B (2015). Adaptive laboratory evolution of ethanologenic *Zymomonas mobilis* strain tolerant to furfural and acetic acid inhibitors. Appl Microbiol Biotechnol.

[CR99] Silveira MM, Wisbeck E, Lemmel C (1999). Bioconversion of glucose and fructose to sorbitol and gluconic acid by untreated cells of *Zymomonas mobilis*. J Biotechnol.

[CR100] Smith KM, Cho KM, Liao JC (2010). Engineering *Corynebacterium glutamicum* for isobutanol production. Appl Microbiol Biotechnol.

[CR101] Snoep JL, Arfman N, Yomano LP (1994). Reconstitution of glucose uptake and phosphorylation in a glucose-negative mutant of *Escherichia coli* by using *Zymomonas mobilis* genes encoding the glucose facilitator protein and glucokinase. J Bacteriol.

[CR102] So LY, Chen WY, Lacap-Bugler DC (2014). PZMO7-Derived shuttle vectors for heterologous protein expression and proteomic applications in the ethanol-producing bacterium *Zymomonas mobilis*. BMC Microbiol.

[CR103] Sootsuwan K, Thanonkeo P, Keeratirakha N (2013). Sorbitol required for cell growth and ethanol production by *Zymomonas mobilis* under heat, ethanol, and osmotic stresses. Biotechnol Biofuels.

[CR104] Sulfahri AM, Sumitro SB, Saptasari M (2016). Bioethanol production from algae *Spirogyra hyalina* using *Zymomonas mobilis*. Biofuels.

[CR105] Swings J, De Ley J (1977). The biology of *Zymomonas*. Bacteriol Rev.

[CR106] Tani Y, Vongsuvanlert V (1987). Sorbitol production by a methanol yeast, *Candida boidinii* (Kloeckera sp.) No. 2201. J Ferment Technol.

[CR107] Taran M, Lotfi M, Safaei M (2019). Optimal conditions for levan biopolymer production and its use in the synthesis of bactericidal levan-zno nanocomposite. Biotechnologia.

[CR108] Taştan Ö, Sözgen G, Baysal T, Kaplan Türköz B (2019). Production of prebiotic 6-kestose using *Zymomonas mobilis* levansucrase in carob molasses and its effect on 5-HMF levels during storage. Food Chem.

[CR109] Todhanakasem T, Sangsutthiseree A, Areerat K (2014). Biofilm production by *Zymomonas mobilis* enhances ethanol production and tolerance to toxic inhibitors from rice bran hydrolysate. N Biotechnol.

[CR110] Todhanakasem T, Wu B, Simeon S (2020). Perspectives and new directions for bioprocess optimization using *Zymomonas mobilis* in the ethanol production. World J Microbiol Biotechnol.

[CR111] Vasan TP, Piriya SP, Prabhu IGD, Vennison JS (2011). Cellulosic ethanol production by *Zymomonas mobilis* harboring an endoglucanase gene from Enterobacter cloacae. Bioresour Technol.

[CR112] Viikari L, Berry DR (1988). Carbohydrate metabolism in *Zymomonas*. Crit Rev Biotechnol.

[CR113] Wang GJ, Wang ZS, Zhang YW, Zhang YZ (2012). Cloning and expression of *amyE* gene from *Bacillus subtilis* in *Zymomonas mobilis* and direct production of ethanol from soluble starch. Biotechnol Bioprocess Eng.

[CR138] Wang H, Cao S, Wang WT, Wang KT, Jia X (2016). Very high gravity ethanol and fatty acid production of *Zymomonas mobilis* without amino acid and vitamin. J Ind Microbiol Biotechnol.

[CR114] Wang X, He Q, Yang Y (2018). Advances and prospects in metabolic engineering of *Zymomonas mobilis*. Metab Eng.

[CR115] Wang W, Wu B, Qin H (2019). Genome shuffling enhances stress tolerance of *Zymomonas mobilis* to two inhibitors. Biotechnol Biofuels.

[CR116] Weir PM (2016). The ecology of *Zymomonas*: a review. Folia Microbiol (praha).

[CR117] Wirawan F, Cheng CL, Lo YC (2020). Continuous cellulosic bioethanol co-fermentation by immobilized *Zymomonas mobilis* and suspended *Pichia stipitis* in a two-stage process. Appl Energy.

[CR118] Woodley JM (2017). Bioprocess intensification for the effective production of chemical products. Comput Chem Eng.

[CR119] Wu Y, Li T, Cao Q (2017). RecET recombination system driving chromosomal target gene replacement in *Zymomonas mobilis*. Electron J Biotechnol.

[CR120] Wu R, Chen D, Cao S (2020). Enhanced ethanol production from sugarcane molasses by industrially engineered: *Saccharomyces cerevisiae* via replacement of the PHO4 gene. RSC Adv.

[CR121] Xia J, Yang Y, Liu CG (2019). Engineering *Zymomonas mobilis* for Robust Cellulosic Ethanol Production. Trends Biotechnol.

[CR122] Xu Y, Chu H, Gao C (2014). Systematic metabolic engineering of *Escherichia coli* for high-yield production of fuel bio-chemical 2,3-butanediol. Metab Eng.

[CR123] Yang S, Fei Q, Zhang Y (2016). *Zymomonas mobilis* as a model system for production of biofuels and biochemicals. Microb Biotechnol.

[CR124] Yang S, Mohagheghi A, Franden MA (2016). Metabolic engineering of *Zymomonas mobilis* for 2,3-butanediol production from lignocellulosic biomass sugars. Biotechnol Biofuels.

[CR125] Yang Y, Shen W, Huang J (2019). Prediction and characterization of promoters and ribosomal binding sites of *Zymomonas mobilis* in system biology era. Biotechnol Biofuels.

[CR126] Yi X, Gao Q, Bao J (2019). Expressing an oxidative dehydrogenase gene in ethanologenic strain *Zymomonas mobilis* promotes the cellulosic ethanol fermentability. J Biotechnol.

[CR127] Yoon KH, Park SH, Pack MY (1988). Transfer of *Bacillus subtilis* endo-β-1,4-glucanase gene into Zymomonasanaerobia. Biotechnol Lett.

[CR128] Zachariou M, Scopes RK (1986). Glucose-fructose oxidoreductase, a new enzyme isolated from *Zymomonas mobilis* that is responsible for sorbitol production. J Bacteriol.

[CR131] Zhang S, Voigt CA (2018). Engineered dCas9 with reduced toxicity in bacteria: Implications for genetic circuit design. Nucleic Acids Res.

[CR129] Zhang M, Eddy C, Deanda K (1995). Metabolic engineering of a pentose metabolism pathway in ethanologenic *Zymomonas mobilis*. Science.

[CR130] Zhang Q, Nurhayati CCL (2017). Ethanol production by modified polyvinyl alcohol-immobilized *Zymomonas mobilis* and in situ membrane distillation under very high gravity condition. Appl Energy.

[CR132] Zhang K, Lu X, Li Y (2019). New technologies provide more metabolic engineering strategies for bioethanol production in *Zymomonas mobilis*. Appl Microbiol Biotechnol.

[CR133] Zhang M, Chou Y-C, Franden MA, Himmel L (2019b) Enginnering *Zymomonas* for the production of 2,3-butanediol. US20190153483A1

[CR134] Zhao N, Bai Y, Liu CG (2014). Flocculating *Zymomonas mobilis* is a promising host to be engineered for fuel ethanol production from lignocellulosic biomass. Biotechnol J.

[CR135] Zheng Y, Han J, Wang B (2019). Characterization and repurposing of the endogenous Type I-F CRISPR-Cas system of *Zymomonas mobilis* for genome engineering. Nucleic Acids Res.

[CR136] Zou SL, Hong JF, Wang C (2012). Construction of an unmarked *Zymomonas mobilis* mutant using a site-specific FLP recombinase. Food Technol Biotechnol.

[CR137] Zou SL, Zhang K, You L (2012). Enhanced electrotransformation of the ethanologen *Zymomonas mobilis* ZM4 with plasmids. Eng Life Sci.

